# Medical Home Care and Educational Services for Children and Youth on the Autism Spectrum: A Scoping Review

**DOI:** 10.1007/s10803-024-06235-3

**Published:** 2024-02-28

**Authors:** Sabrin Rizk, Emmanuel M. Ngui, Zurisadai Salgado, Dianna L. Bosak, Mary A. Khetani

**Affiliations:** 1 School of Rehabilitation Sciences and Technology, University of Wisconsin-Milwaukee, Milwaukee, WI USA; 2 Department of Occupational Therapy, University of Illinois Chicago, Chicago, IL USA; 3 Community and Behavioral Health Promotion, University of Wisconsin-Milwaukee, Milwaukee, WI USA

**Keywords:** Autism spectrum, Primary care, Medical home, Early intervention, Individualized family service plan, Individualized education program

## Abstract

This scoping review examined current evidence on medical home care and its association with educational services for children and youth on the autism spectrum. We searched five databases and grey literature resulting in 328 publications. Publications meeting inclusion criteria were mapped to medical home care component(s) addressed, type(s) of educational services and their strength and type of association. The Andersen Behavioral Model of Health Services Use was used to summarize predisposing, enabling, and need factors considered. Eighteen publications were reviewed, including eight practice/policy reports and ten original research publications. Medical home care components most addressed included family-centered care (n = 10), referrals (n = 16), and effective care coordination (n = 13). Seven publications also addressed multiple educational service types. Two of the five publications that established a significant association between medical home care components and educational services had mixed results, with one publication reporting a negative association and the other publication reporting a positive association. Challenges to medical home care and educational services were most categorized as enabling factors. Results suggest three areas for further investigation: (1) limited evidence on the strength and type of association between medical home care components and educational services; (2) limited use of population data sources; and (3) the need to consider a broader range of factors when examining their association.

## Introduction

Families of children and youth on the autism spectrum (CYAS) deserve equitable access to multiple service systems, including medical home care and educational services. The American Academy of Pediatrics (AAP) endorses a medical home care model that emphasizes use of a single, centralized source of primary care, (AAP, 2002). CYAS can benefit from equitable access to quality medical home care due to their extensive need for multiple specialized health-related services (e.g., neurology, gastroenterology, behavioral health, therapeutic services) beyond well-child visits AAP, 2002). However, CYAS are four times more likely to have unmet health care needs (Karpur et al., 2016) and to lack certain medical home components (e.g., care coordination) (Sobotka et al., 2016) and family-centeredness, resulting in decreased satisfaction with care (Al-Mazidi & Al-Yaaadi, 2021). CYAS lacking medical home care are more likely to have unmet service need, greater dissatisfaction with their care, and poor health outcomes (Cheak-Zamora & Farmer, 2015; Farmer et al., 2010).

In addition to navigating medical home care, it is common for CYAS to encounter the educational service system sooner than their peers (Shahidullah et al, 2018), especially for children who are diagnosed as early as 18 months old (Lord et al., 2006). CYAS and their families commonly navigate Individuals with Disabilities Education Act (IDEA) Part C early intervention (EI) or Part B special education services in public school systems. Educational settings can situate medical home care functions within the school-based setting, such as school-based health centers located in low-income, under-resourced, urban settings and involve partnership between a public school and community health center, hospital or local health department. School-based health centers can increase families’ access to cross-sector care involving medical home care and educational services (Badgett et al., 2022; O’Leary et al., 2019). However, school-based health centers may not be equipped to adequately meet the complex needs of CYAS and their families (Williams et al., 2012) comparable to standard medical home care capacity or national medical home recognition parameters (Gregg et al., 2019).

Families typically assume primary responsibility for navigating medical home care and educational services (Adams & Tapia, 2013; Dunn et al., 2016; Lipkin & Okamoto, 2015). Most communications between medical home providers and school-based programs are done through families with limited support for families when communicating complex issues with schools (Russell & McCloskey, 2016). Families are thereby tasked to navigate multiple professional specialties with different governing entities, multiple entry points, and distinct lexicons that can challenge their access to medical and educational services (Dunn et al., 2016). Families of CYAS must grapple with the use of diagnostic criteria for autism, which is guided by the Diagnostic and Statistical Manual of Mental Disorders (DSM) (Lobar, 2016). Simultaneously, they have to understand the educational classification of autism in public schools, which is based on the Individuals with Disabilities Education Act (IDEA) Part B (Smith, 2005a, 2005b). These are two distinct systems with their own unique terminologies and criteria, adding to the challenge families face. Similarly, families may encounter health professionals in an educational service system (e.g., occupational therapy, speech therapy, and physical therapy) who prescribe specialized therapies that are educationally relevant (Adams & Tapia, 2013). Other educationally-related services provided in school settings do not require insurance coverage, which often require referrals, if services are deemed reimbursable.

The Andersen Behavioral Model of Health Services Use (BMHSU), initially for health services, will be used to assess educational services for CYAS via the medical home pathway (Andersen et al., 2013). The theory posits that health care service use is a function of three factors: predisposing, enabling, and need factors. Predisposing factors are considered to increase the propensity health service use in individuals and families. Enabling factors can either promote or impede the service use. Need factors suggests that services are used in response to functional and health problems.

Despite its potential, the model’s application in educational service use is limited. One study investigated the relationship between the ease of access to community-based and educational services and the need factors among children and youth with special health care needs, developmental delay, and intellectual disability. It found that nearly half (49.7%) of children with developmental conditions encountered difficulties in accessing services, with 16.9% lacking access to educational services. Factors such as functional limitations, care coordination needs, the type of developmental condition, and early intervention significantly influenced their use of educational services (Lindly et al., 2015). Another study using the Andersen BMHSU and the 2012 National Survey of Children’s Health to assessed health care disparities among racially and ethnically diverse school-aged children (6–17 years) with health conditions, and their access to an individualized education program (IEP). The ABMHSU was successful in predicting the likelihood of having an IEP among children with health conditions. Notably, Hispanic and Black, non-Hispanic children were less likely than White, non-Hispanic children to have an IEP. However, Black, non-Hispanic, Hispanic, and Multiracial children with increased family and neighborhood resources and greater health needs were more likely to have an IEP (Hinojosa et al., 2017).

To comprehensively grasp cross-sector care for families of CYAS and pinpoint areas that require further research, there is need to summarize existing evidence regarding their access to medical home care and educational services. We carried out this scoping review to determine what the existing body of evidence reveals about the relationship between medical home care and educational services in CYAS. The aims of this scoping review are to characterize: (1) the association between medical home care and educational services for CYAS (Aim 1), and (2) the predisposing, enabling, and need factors, according to ABMHSU, that attenuate or intensify the association between medical home care and educational services (Aim 2).

## Methods

**Fig. 1:**
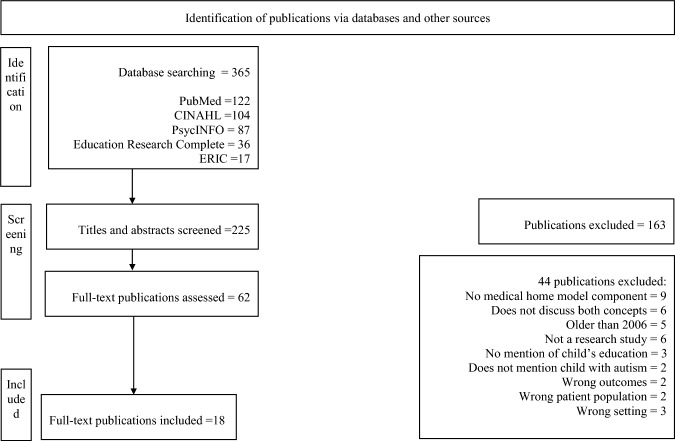
PRISMA Flowchart detailing databases searched, including other sources, and the number of abstracts and full text retrieved

To guide our scoping review, we used a five-step process (Arksey & O’Malley, 2005) and supplemented with other prominent scoping review guidelines (Arksey & O’Malley, 2005; Aromataris & Munn, 2020; Levac et al., 2010; Peters et al., 2021) to identify available evidence on the association between medical home care and educational services for CYAS. The scoping review protocol was developed a priori and registered prospectively with the Open Science Framework (Rizk & Khetani, 2021). For this review, we operationalized medical home care consistent with the five medical home care components (i.e., personal doctor or nurse, usual source of sick care, family-centered care, getting referrals, and effective care coordination) from the National Survey of Children’s Health (NSCH) (U.S. Census Bureau, 2018). We operationalized educational services as the receipt of services under an individualized family service plan (IFSP) and individualized education program (IEP) (Smith, 2005a, 2005b). We used the PRISMA-ScR checklist (see Fig. 1) from the Joanna Briggs Institute (JBI) Manual for Evidence Synthesis (Aromataris & Munn, 2020).

### Step One: Identifying the Research Question

The research questions were: (1) What is known about medical home care and its association with educational services for CYAS? and (2) What factors are associated with medical home care and educational services in CYAS?

### Step Two: Identifying Relevant Studies

The first author (SR) performed comprehensive literature searches of electronic bibliographic databases in PubMed, CINAHL Plus Full Text, PsycINFO, ERIC, and Education Research Complete to identify publications that met inclusion criteria for full. Additionally, the reference lists of all included publications were hand searched to ensure relevant literature were identified. The first author (SR) performed a grey literature search in Google Scholar to locate and scan reference lists of policy statements published in *Pediatrics*.

The first author (SR) received assistance from a health sciences reference librarian with experience in scoping review methodology to conduct the literature search. Publications were limited to those published between 2006 through December 2022. The 2006 selection was guided by the AAP's release of an algorithm for developmental monitoring in primary care, targeting early detection and EI referral and establish rules to enhance monitoring and screening of children and youth with disabilities in medical home care. The algorithm includes autism-specific screening at 18–24 months to facilitate early detection and referral of children at risk or diagnosed with autism for detailed evaluations and potential early intervention (EI) (AAP; 2006; AAP, Council on Children with Disabilities, Section on Developmental Behavioral Pediatrics, Bright Futures Steering Committee, 2006).

We searched key terms related to autism, medical home care, and educational services. These key terms were refined and broadened per scoping review guidelines (Arksey & O’Malley, 2005; Aromataris & Munn, 2020; Levac et al., 2010; Peters et al., 2021). ‘Autism’ was broadened to include ‘ASD,’ ‘autistic,’ and ‘Asperger.’ ‘Medical home care’ was broadened to include ‘primary care.’ ‘Educational services’ was broadened to include ‘education,’ ‘IEP,’ ‘IFSP,’ ‘individualized education plan,’ ‘individualized family services plan,’ ‘early intervention,’ and ‘educational.’

### Step Three: Study Selection

Five database searches yielded 365 publications: PubMed (n = 122), CINAHL (n = 104), PsycINFO (n = 87), ERIC (n = 17), and Education Research Complete (n = 36). Ten additional publications were identified through backward and forward reference and citation searching of publications retrieved from a grey literature search (i.e., Google Scholar). The first author (SR) screened titles and abstracts to assess whether publications addressed autism, medical home care, or educational services, including key and/or broadening terms. Publications were included if they met these criteria: (1) children or youth diagnosed with autism spectrum disorder, ASD, Asperger’s syndrome, autistic disorder, or autism based on clinical or parent-reported data, (2) between the ages of 1–17 years old, (3) studies that are available in English language, (4) conducted in the US, (5) published between 2006 to December 2022, and (6) could include policy and position statements. Our study targeted children and youth on the autism spectrum ages 1 to 17 years old, in accordance with national surveys like the National Survey of Children's Health that typically use caregiver-reported data on children ages 0–17 and their families. We excluded publications that focused solely on health and/or medical services or educational services in CYAS, but not both, because they would not be examining how these two service systems work together. The search of the five databases and grey literature resulted in 365 publications screening, and after duplicates were removed, 225 publications remained for title and abstract screening completed by the first author (SR). Both authors piloted inclusion criteria by independently reviewing (Aromataris & Munn, 2020) 10 of the 62 publications. Since no discrepancies were noted during the pilot phase, and no adjustments were made to the inclusion criteria, both authors proceeded to independently perform full text reviews of the remaining 52 publications. The first author (SR) independently reviewed 15 publications. During full text review, 44 of 62 publications were excluded. The first and third authors (SR, ZS) met to discuss and resolve conflicts on the rationale for excluding (see Table 3 in Appendix) 10 of these publications. A total of 18 publications met inclusion criteria based on title and abstract screening and full text review (see PRISMA flowchart (Fig. 1)).

### Step Four: Charting the Data

For Aim 1, the first author (SR) extracted relevant characteristics for included publications: author(s), publication year, study purpose, study design, respondent type, sample characteristics (e.g., child sex, child age, child race/ethnicity, caregiver education level, child insurance type) medical home care component(s) addressed, type(s) of educational service, type of association (positive, negative, or no association) between medical home care and type(s) of educational services.

### Step Five: Collating, Summarizing, and Reporting the Results

For Aim 2, two authors (SR, DB) collated and created a summary of factors attenuating the relationship between medical home care and educational services, according to the Andersen Behavioral Model of Health Services Use (BMHSU) (Andersen, 1968). Predisposing factors are individual or family traits that can affect their likelihood to use services. These factors include demographics (e.g., age or ethnicity), and sociocultural elements (e.g., beliefs and health knowledge). Enabling factors are conditions that affect the service use. They can include service availability and accessibility, financing, and the existence of social support or health care infrastructure. Need factors are recognized or assessed health issues that require the service use. In collating the content from the included publications, we identified patterns and explanations that described the factors attenuating the relationship between medical home care and educational services. The first and fourth authors (SR, DB) began with creating themes to broadly capture the type of factors most cited in the available evidence and sorted the various factors under each of the four themes (see Table 2). After sorting specific factors according to type, the first author (SR) categorized each predisposing, enabling, and need factor using the Andersen BHMSU. The first and fourth authors (SR, DB) then discussed the sorting process, and discrepancies noted in the “Provider Training, Knowledge, Awareness, and Skills” and “Organizational Resources and Workflows” were discussed until we reached consensus.

Included publications were summarized using descriptive statistics (frequency counts and percentages) to provide an overview of available evidence on medical home care in relationship to educational services, considering differences in predisposing, enabling, and need factors.

## Results

### Characteristics of Included Publications

Table 1 illustrates that the 18 included publications across multiple formats. Eight original publications used cross-sectional (n = 4; 22%), prospective cohort (n = 1; 5%), mixed-method (n = 1; 5%), randomized controlled trial (n = 1; 5%), or qualitative (n = 1; 5%) study designs. One original research publication utilized secondary analyses of a population-based data source (i.e., National Survey of Children with Special Health Care Needs) (Sobotka et al., 2016).

**Table 1:** Characteristics of included publications

Author(s), year	Study design	Population	Medical home care component(s)					Educational service type		Type of association			
			PDN	USC	FCC	Ref	ECC	EI	SPED	N/A	+	None	−
Aloisio and Huron (2020)	Review publication	N/A			X	X	X	X	X	X			
Buranova et al. (2022)	Commentary	NA			X	X	X	X		X			
Carbone et al. (2010)	Qualitative	Caregivers of children on autism spectrum (n = 5); Pediatricians (n = 9)			X	X	X	X	X	X			
Council on Children with Disabilities et al. (2006)	Policy statement	N/A			X	X	X	X	X	X			
Dang et al. (2017)	Prospective cohort	Caregivers of children on autism spectrum (n = 30)					X		X			X	
Dick et al. (2021)	Cross-sectional	Caregiver of children on autism spectrum (n = 37) or DEV (n = 22)			X			X				X	
Duby (2007)	Policy statement	N/A			X	X	X	X	X	X			
Edwards et al. (2021)	Cross-sectional	Physicians or primary care providers (n = 99)					X	X				X	
Ellerbeck et al. (2015)	Practice report	N/A				X	X		X	X			
Hyman et al. (2020)	Clinical report	N/A			X	X	X		X	X			

Author(s), year	Study design	Respondent type	Medical home care component(s)					Educational service type		Type of association			
			PDN	USC	FCC	Ref	ECC	EI	SPED	N/A	+	None	−
Ibañez et al. (2019)	Randomized controlled trial	Primary care providers (n = 58), EI providers (n = 87), ASD Concerns (n = 65), Developmental delay (n = 68), No Concerns (n = 184)				X		X		X			
Lobar (2016)	Practice report	N/A				X	X		X	X			
McClain et al. (2020a, 2020b)	Mixed Methods	School psychologists (n = 203)					X		X		X		
Shahidullah et al. (2018)	Practice report	N/A			X	X	X	X	X	X			
Shahidullah et al. (2020)	Practice report	N/A					X		X	X			
Sobotka et al. (2016)	Cross-sectional	Children on autism spectrum (n = 2859)	X	X	X		X	X	X				X
Sohl et al. (2022)	Opinion	NA				X		X		X			
Williams et al. (2012)	Cross-sectional	Parents of children on autism spectrum (n = 114); Physicians (n = 125)	X	X	X	X	X		X			X	

PDN = Personal doctor or nurse, USC = Usual source of care, FCC = Family centered care, Ref = Referrals, ECC = Effective care coordination, EI = Early intervention, SPED = Special education, N/A = Not applicable to measure type of association based on study design, +  = positive association, − = negative association, None = no association

Original publications that focused on children and youth samples who were predominantly male (Carbone et al., 2010, 2016; Dick et al., 2021; Sobotka et al., 2016), with age ranges spanning from 2 to 35 years old (Carbone et al., 2010, 2016; Dick et al., 2021; Williams et al., 2012). Most caregivers were White, non-Hispanic (Carbone et al., 2010; Dick et al., 2021; Sobotka et al., 2016; Williams et al., 2012). Most CYAS held private insurance and belonged to households where English was the primary language (Carbone et al., 2010). Moreover, their caregivers had an education level that extended beyond high school. While the study by Williams et al. (2012) had a higher age limit compared to ours, most of our age categories were included within their selection parameters.

In the original research publications involving health care providers, these respondents included physicians or primary care providers (e.g., pediatricians, family medicine physicians, nurse practitioners) (Carbone et al., 2016; Dang et al., 2017; Edwards et al., 2021; Sohl et al., 2022; Williams et al., 2012), and caregivers of CYAS (Carbone et al., 2010; Dang et al., 2017; Dick et al., 2021; Sobotka et al., 2016). In those original research publications involving educational service professionals, respondents included EI providers (Ibañez et al., 2019), early child educators or teachers (Buranova et al., 2022; Dang et al., 2017), school psychologists (McClain et al., 2020a, 2020b), and skilled therapists (e.g., speech and language pathologists, occupational therapists) (Buranova et al., 2022)).

### Characteristics of Medical Home Care and Educational Service Type(s) (Aim 1)

In 18 of the included publications, there is an implicit or explicit focus on one or more components of medical home care. Per the NSCH definition of medical home care, these publications captured the components of experiencing a personal doctor or nurse (n = 2; 11%) (Sobotka et al., 2016; Williams et al., 2012), usual source of sick care (n = 2; 11%) (Sobotka et al., 2016; Williams et al., 2012), family-centered care (n = 10; 56%) (Aloisio et al., 2020; Buranova et al., 2022; Carbone et al., 2010; Dick et al., 2021; Duby, 2007; Hyman & Johnson, 2020; Shahidullah et al., 2020; Sobotka et al., 2016; Williams et al., 2012) referrals (n = 16; 89%) (Aloisio et al., 2020; Buranova et al., 2022; AAP, Council on Children with Disabilities et al., 2006; Dick et al., 2021; Duby, 2007; Edwards et al., 2021; Hyman & Johnson, 2020; Shahidullah et al., 2018; Williams et al., 2012) and effective care coordination (n = 13; 82%) (Aloisio et al., 2020; Carbone et al., 2010; AAP, Council on Children with Disabilities et al., 2006; Dang et al., 2017; Duby, 2007; Ellerbeck et al., 2015; Lobar, 2016; McClain et al., 2020a; Sobotka et al., 2016; Shahidullah et al., 2018, 2020; Williams et al., 2012) (See Table 1).

Similarly, all 18 included publications focus on one or both educational service type(s). Seven of the 18 (39%) included publications examined educational services inclusive of both EI and special education, whereas 7 publications solely examined EI services (39%), and four publications solely examined special education services (22%) (see Table 1). Nine of the 18 included publications addressed multiple medical home care components and both EI and special education service types (Aloisio et al., 2020; Buranova et al., 2022; Carbone et al., 2010; AAP, Council on Children with Disabilities et al., 2006; Duby, 2007; Edwards et al., 2021; Shahidullah et al., 2020; Sobotka et al., 2016; Sohl et al., 2022) (See Table 1).

### Association Between Medical Home Care and Educational Services (Aim 1)

As shown in Table 1, five studies have highlighted associations between the medical home care components and educational services. Among these studies, one demonstrated a negative association, while another indicated a positive one.

### Factors Attenuating or Intensifying the Association Between Medical Home Care and Educational Services (Aim 2)

As shown in Table 2, there was 1 predisposing factor and 12 enabling factors each described as attenuating the association between medical home care component(s) and educational services for CYAS and their families (Andersen et al., 2013). Prior publications have not cited need factors as attenuating or intensifying the relationship between medical home care component(s) and educational services for this population.

**Table 2:** Factors attenuating the relationship between medical home care and educational services

	Predisposing	Enabling	Need
Child and family demographics			
Access to services and resources dependent on social conditions	X		
Provider training, knowledge, awareness, and skills			
Lack of adherence to AAP guidelines on autism surveillance and screening		X	
Hesitancy to discuss early autism concerns and responsiveness to caregivers’ concerns about their children’s development		X	
Lack of knowledge of autism and complex needs		X	
Lack of knowledge on how to direct caregivers to educational services (e.g., referral, service eligibility criteria)		X	
Organizational resources and workflows			
Limited guidelines on how to coordinate between medical home care, EI, and schools		X	
Transitions into and out of educational services (i.e., EI, school, post-secondary)		X	
Insufficient provider availability (i.e., high volume of service need, limited supply of service providers)		X	
Lack of provider time		X	
Long diagnostic and service wait times		X	
Lack of reimbursement for care coordination		X	
Health policies			
Different organizational and/or structural characteristics to fund, treat, and document		X	
Variable state and local laws related to service eligibility and provision		X	

Select child and family demographic characteristics (Andersen et al., 2013) that have so far been included for their potential role in predisposing families to inequitable access to medical home care component(s) and educational services include: (1) primary household language (Sobotka et al., 2016), (2) child race/ethnicity (Dick et al., 2021; McClain et al., 2020a; Sobotka et al., 2016), (3) caregiver education level (Dick et al., 2021; McClain et al., 2020a), and (4) geographic region (Buranova et al., 2022; Edwards et al., 2021). Caregivers who were non-Hispanic White mothers with higher education levels, and older children with private insurance, were significantly less likely to report receiving help as they needed with care coordination, inclusive of doctors’ communication with their child’s school, EI program, or other educational setting (Sobotka et al., 2016). Additionally, families who reside in rural areas lack access to adequate services due to the limited number of providers available to aid children, youth, and families (Buranova et al., 2022; Edwards et al., 2021).

Three levels of enabling factors were included in prior publications for their hypothesized role in attenuating the relationship between medical home care component(s) and educational services for CYAS and their families (McClain et al., 2020a; von Lengerke et al., 2013). At the provider level, lack of adherence to AAP guidelines on autism surveillance and screening (AAP, Council on Children with Disabilities et al., 2006; Hyman & Johnson, 2020; Shahidullah et al., 2018; von Lengerke et al., 2013), hesitancy to discuss early autism concerns and responsiveness to caregivers’ concerns about their children’s development (AAP, Council on Children with Disabilities et al., 2006; Carbone et al., 2016; Edwards et al., 2021) lack knowledge of autism and complex needs, lack of knowledge on how to direct caregivers to educational services (e.g., referral, service eligibility criteria) were regularities cited as attenuating factors (Carbone et al., 2010; Dick et al., 2021; Sobotka et al., 2016; Shahidullah et al., 2018; Lobar, 2016; von Lengerke et al., 2013). At the program level, limited guidelines on how medical home care and educational service should coordinate (Shahidullah et al., 2018), transitions that are specific to the educational setting (i.e., EI, school, post-secondary) (Carbone et al., 2010; Hyman & Johnson, 2020), insufficient provider availability to meet the volume of service needs, lack of medical home care providers’ time to manage the coordination, long diagnostic and service wait times, and reimbursement for providers’ time expended on coordination were factors that limited organizational capacity to facilitate coordination with educational settings (Carbone et al., 2010; Dick et al., 2021; Shahidullah et al., 2018; Sobotka et al., 2016; Williams et al., 2012). At the service system level, differences in organizational or structural characteristics (i.e., funding, intervention, and documentation) and various local and state laws related to service eligibility (Hyman & Johnson, 2020; McClain et al., 2020b), and provision acted as barriers to medical home care and educational services (Aloisio et al., 2020; Carbone et al., 2010; Shahidullah et al., 2018). Privacy and confidentiality policies specific to medical home care (i.e., HIPAA) and to educational settings (i.e., FERPA) were cited as attenuating factors to cross-sector care (McClain et al., 2020a).

## Discussion

This scoping review examines current evidence regarding the association between medical home care and educational services, and the factors that are known to attenuate or intensify this association, for CYAS. Despite the clear benefits of offering integrated medical and educational care, there is not enough of it. Based on the findings of this review, the results highlight three areas for further investigation: (1) limited evidence on the association between medical home care component(s) and educational services; (2) limited use of relevant population-based data sources on this topic; and (3) need to consider a broader range of factors associated with medical home care and educational services. Each of these areas is discussed as they guide future research directions.

### Limited Evidence on the Strength and Type of Association Between Medical Home Care and Educational Services

Our first aim was to characterize the association between medical home care and educational services for CYAS. Five original research studies have underscored the relationships between components of medical home care and educational services. Of these studies, one found a negative correlation, while another study showed a positive association (McClain et al., 2020a, 2020b; Sobotka et al., 2016).

This finding can be attributed to the small number of original research publications that we identified, which used a variety of methodologies (e.g., randomized control trial, mixed method approach, secondary analyses) to explore medical home care component(s) in relationship to educational services for CYAS (see Table 1). Alternatively, the five publications that estimated associations did not model all medical home care component(s) in relationship to educational services (see Table 1), preventing a comprehensive measurement of this association and how CYAS access educational services through the medical home care pathway.

In addition, the emphasis on the outcome of educational services, relative to EI, and less with special education, is another future direction. Given that many CYAS access special education services longer, compared to EI (Aloisio et al., 2020; AAP, Council on Children with Disabilities et al., 2006; Carbone et al., 2010; Dick et al., 2021; Duby, 2007; Ibañez et al., 2019; Sobotka et al., 2016), and effective care coordination (n = 13; 82%) (Aloisio et al., 2020; Carbone et al., 2010; AAP, Council on Children with Disabilities et al., 2006; Dang et al., 2017; Duby, 2007; Ellerbeck et al., 2015; Lobar, 2016; McClain et al., 2020a, 2020b; Sobotka et al., 2016; Shahidullah et al., 2018, 2020; Williams et al., 2012) which is a more limited time service, addressing this imbalance will provide a better understanding of how CYAS access special education to determine the extent to which their service needs are being addressed.

### Limited Use of Population-Based Data Sources

There have been several clinical and practice reports published alongside original research studies (cross-sectional, prospective cohort, mixed-method, randomized control trial, qualitative), but only one publication involved secondary analyses of population-based data. We recently determined a negative association between medical home care and educational services for CYAS based on population-based data (Rizk et al., 2023). Studies like this can benefit from data sources that include a robust definition to reflect AAP's intent on how medical home care should be provided (AAP, 2001), for consistent operationalization of the medical home care concept (Child and Adolescent Health Measurement Initiative, 2009). In addition to NSCH and other relevant population-based data sources, other more localized data sources (e.g., electronic health record data) may also be useful to confirm, disconfirm, and/or extend knowledge about the association between medical home care with educational services. Furthermore, it should be noted that the AAP definition is a medical definition and therefore has limitations when used to examine access and use to other services, including educational services.

### Need to Examine Broader Range of Factors Attenuating the Association Between Medical Home Care and Educational Services

We used the Andersen BMHSU (Andersen, 1995; Andersen et al., 2013; Babitsch et al., 2012) to explore the available evidence on predisposing, enabling, and need factors attenuating the access educational services in relation to healthcare utilization (i.e., medical home care). We found that enabling factors were the most salient in examining the association. In contrast, we found limited evidence on the role of predisposing factors, and no evidence on the role of need factors, as attenuating or intensifying the association between medical home care and educational services.

Enabling factors (Andersen et al., 2013) captured reflect the distinguished and unique features of medical home care and educational services, in terms of their service intent, design, and delivery. Based on the Andersen BMHSU, we identified three categories of enabling factors: (1) provider training, knowledge, awareness, and skills; (2) organizational resources as impacting provider workflows; and (3) health and educational policies (see Table 2).

A first type of enabling factor relates to provider training, knowledge, awareness, and skills to discuss autism and its associated comorbidities (e.g., attention difficulties, anxiety, sensory issues, sleep disorders) (Aloisio & Huron, 2020; Williams et al., 2012). In Williams et al.'s study, more than half of the sampled physicians had worked more than 15 years in practice. There were many physicians who have never attended a Continuing Medical Education on autism. Without ongoing training, physicians will be unable to meet even the basic needs of children, youth, and their families. Edwards et al. (2021) examined provider hesitancy to discuss early autism concerns and responsiveness to caregivers’ concerns about their children’s development. Current evidence suggests that medical home care providers can increase caregiver awareness of educational services, respond to caregiver concerns, and adhere to recommended autism screening timelines. (Carbone et al., 2010; AAP, Council on Children with Disabilities et al., 2006; Duby, 2007; Ellerbeck et al., 2015; Hyman & Johnson, 2020; Ibañez et al., 2019; Sobotka et al., 2016; Williams et al., 2012).

A second type of enabling factor relates to several organizational resources as impacting workflow, including lack of provider time, long diagnostic and service wait times, and workforce capacity with insufficient provider availability (Aloisio et al., 2020; Buranova et al., 2022; Carbone et al., 2010; Dick et al., 2021; Duby, 2007; Edwards et al., 2021; Ellerbeck et al., 2015; Hyman & Johnson, 2020; McClain et al., 2020b; Sobotka et al., 2016; Shahidullah et al., 2018). The AAP (2006) recommends well-child visits every month until approximately 2 years of age, then yearly or as needed after that, whereas EI providers often see families weekly (Dick et al., 2021). Carbone et al. (2010) observed a shortage of physicians who feel qualified to care for CYAS and other developmental impairments and would benefit from more resources (e.g., family groups and schools) to support medical home care. For CYAS, resource limitations may lead to infrequent and brief interactions with primary care providers. A significant majority of primary care providers (85%) recognized that time constraints are the principal challenge when caring for this demographic. A smaller group (17%) noted the need for devoting more time for patient interaction and advocating for administrative accommodations, such as specialized appointment slots for CYAS. Simultaneously, providers understand that spending more time with CYAS and their families to ensure adequate care necessitates appropriate compensation. To this end, 8% of providers emphasize the potential benefits of improved reimbursement strategies and a comprehensive understanding of billing and coding systems. This would ensure that the additional time they spend with CYAS and their families is adequately compensated (Mazurek, et al., 2020).

Differences in health and educational policy are a third type of enabling factor. These policy differences may be related to service eligibility (e.g., DSM-V versus IDEA) (Ellerbeck et al., 2015; Hyman & Johnson, 2020; Lobar, 2016), such as differences in autism medical diagnostic criteria and autism educational criteria used to determine eligibility for special education services (McClain et al., 2020a, 2020b; Hyman & Johnson, 2020). Financing barriers include reimbursement for coordination efforts and longer health care visit durations (Carbone et al., 2010), where the time required by primary care providers to coordinate care and consult with other services may exceed what insurance reimburses (Dick et al., 2021). Furthermore, educational services are supplied at no cost to families (Hyman & Johnson, 2020)), as opposed to health-care costs that rely on third-party payers (e.g., health-care systems) (Ellerbeck et al., 2015). Both service systems have their own unique documentation processes. For example, electronic health records are utilized in the health care system (Aloisio & Huron, 2020; Ellerbeck et al., 2015), whereas educational service plans are outlined in IFSPs or IEPs (Ellerbeck et al., 2015; Hyman & Johnson, 2020).

Research on predisposing and need factors is needed when examining the association between medical and educational services for CYAS (Babitsch et al., 2012; Andersen et al., 2013). The absence of need factors can be explained by the fact that medical home providers vary in their training, and this may hinder their ability to accurately capture important need factors, such as condition severity (Duby, 2007) or address many of the comorbid conditions (e.g., attention deficits, anxiety, sensory issues, sleep disorders), associated with autism (Aloisio & Huron, 2020; Williams et al., 2012). Similarly, we contend that need factors, while important, were muted in these studies possibly because “need” is implied with CYAS. These children and youth may have other additional health and social needs that should be monitored and addressed through better cross-sector care. Most of the factors in this review that have been shown to attenuate the relationship between medical home care and educational service use are also factors that if addressed can increase cross-sector care between medical home care and educational services.

There is a possibility that the relationship between medical home care and educational services is indirect, such as by means of medical home care referrals to allied health services provided in school rather than in hospitals or clinics. Referring patients and families to specialists based on positive or elevated screening scores is within a primary care provider’s scope of practice (AAP, 2001), and this practice is further endorsed by IDEA Subpart D that defines primary referral sources to educational services as including hospitals, physicians and other clinics and health care providers (U.S. Department of Education, 2017) (p. 14, lines 14–22). If future studies measured all medical home care components (e.g.., referral), we may test more specified hypotheses about its association with educational services. For example, it would be possible to examine potential indirect links between medical home care referral to educational services by another mitigating factor (e.g., use of specialized therapies).

This review has four key limitations. First, it is possible that we have missed publications that did not mention medical home care or educational services in their title or abstract. Second, data extraction from included publications was performed independently by the first author (SR), which could have resulted in incorrect data classification. Third, results are limited to children and youth ages 1–17 years old and do not generalize to transition-aged youth on the autism spectrum who may be continuing to receive medical home primary care through their pediatrician as part of their transition to adult health care services. Fourth, the Andersen BMHSU has traditionally been used to measure access and use of health care services based on predisposing enabling, and need factors (Andersen et al., 2013). We explored how it could be applied to other services, such as educational services, as part of our review. Our review included 18 publications, of which five studies have underscored the relationships between components of medical home care and educational services. Of these five studies, one found a negative correlation, while another study showed a positive correlation. This scoping review provides new insights into the topic of medical home care as it pertains to educational services. The results pinpoint three areas that warrant further investigation: (1) limited evidence on the association between medical home care component(s) and educational services; (2) limited use of relevant population-based data sources on this topic; and (3) need to consider a broader range of factors associated with medical home care and educational services. Each of these areas is discussed as they guide future research directions. In addition to the present study, subsequent studies using population-based data sources will further examine medical home care on a granular level to identify the component(s) of medical home care that are closely related to educational services as well as factors attenuating their association for CYAS.

## Appendix

See Table 3.

**Table 3:** Reasons for exclusion and corresponding operational definitions

Reason for exclusion	Operational definition
Does not discuss both concepts	Contains no information regarding medical home care and educational services
No medical home component	Neither describes nor includes any of the components of a medical home
Not a research study	Research not published in a refereed journal or not empirical
Older than 2006	Publications published before 2006
No mention of child’s education	Either early intervention or special education services must be included
Wrong outcome	No investigation of the relationship between access to medical home care and use of educational services, such as early intervention or special education, in this document
Wrong setting	The study was not conducted in the United States
Does not mention child with autism	Children with disabilities cannot be regarded as a single population
Wrong patient population	Samples of adults with autism cannot be included
Wrong comparator	There can be no comparison between children and youth on the autism spectrum and neurotypical children
